# First District Medical Society of Ohio

**Published:** 1829

**Authors:** 


46. *First District Medical Society, of Ohio.*—The annual meeting of the Society, was held in this city on the 26th of May. As most of the business was local, we shall notice but two or three of its acts.

CAPITATION TAX ON PHYSICIANS AND LAWYERS.

Two or three years ago, the General Assembly, in their wisdom, levied a poll tax on the members of these two ancient and honorable professions. It could not be less than five, nor more than fifty dollars *a head*, according to the good pleasure of the Court of Common Pleas, of each county. Here was a genuine case of *legal* and *forensic* medicine; and the consultants were drawn from both professions. The gentlemen of the Bar pronounced the law *unconstitutional*, and they of the faculty declared, that it would seriously impair the integrity of the *constitution;* so both parties agreed, that the interests of liberty, not less than those of the two learned professions, demanded its immediate abrogation. To effect this, the lawyers *demurred*, and the doctors advised that the *sphincter bursæ* of every individual, should be kept in a state of constriction. But all would not do. The Supreme Court declared the law constitutional, and the *stomach pump* of the sheriff has already found its way to the bottom of many a purse, which had been firmly closed upon a sum scarcely large enough to satisfy the original demand with the interest which had accumulated upon it. In this *critical stage* of the disorder, the *vis medicatrix* of the first District Society, has again re-acted, and given birth to the following resolution, which we seriously recommend to the attention of all who are interested.

"*Resolved*, That a Committee of seven members be appointed to draft and present to the next General Assembly, a remonstrance against the *statute* imposing an annual tax on the physicians of the state, and a memorial proposing for a repeal of the same; and that they be instructed to request the co-operation of the other District Societies recommending that they transmit their memorials to the President of the State Society, to be by him laid before the General Assembly: Whereupon, Doctors Drake, Warren, Hunt, J. H. Brower, Hough, Wayland, and Hopkins were elected said Committee."

### Resolution against Intemperance.

"On motion of Dr. Warren, it was *unanimously resolved*, That the Society view, with deep regret, the prevalence of an intemperate use of spirituous liquors, and their baneful effects upon the human system; and do hereby pledge themselves to use every exertion in their power to suppress intemperance, both by precept and example, whenever opportunity offers."

### Officers of the Society for 1829.

Daniel Drake, M. D. *President*,
Edwin A. Atlee, M. D. *Vice President*,
Vincent C. Marshall, M. D. *Corresponding Secretary*,
James Warren, M. D. *Recording Secretary*,
Melancthon Rogers, M. D. *Treasurer*,
Isaac Hough, M. D. *Librarian*,

Doctors Vincent C. Marshall, John Woolley, William Wayland, Jeremiah H. Brower, and Isaac Hough, *Censors*.

